# ICU mortality rates in patients with sepsis compared with patients without sepsis

**DOI:** 10.1186/cc14094

**Published:** 2015-03-16

**Authors:** J Melville, S Ranjan, P Morgan

**Affiliations:** 1 East Surrey Hospital, Redhill, UK

## Introduction

The aim of the study was to evaluate the difference in mortality rates between those admitted to the ICU with and without sepsis, and to assess the proportion of patients who had sepsis. Septic patients are one of the key groups of patients admitted to ICUs around the world. Septic patients have an extremely poor prognosis with published mortality rates ranging from 20.7% (severe sepsis) to 45.7% (septic shock) [1]. With septic patients making up roughly 21% of patients admitted to ICUs, it is important to assess whether these rates of mortality hold true to a district general ICU and to assess the extent of the difference in prognosis between patients with and without sepsis [2].

## Methods

We performed a retrospective case note review, looking at a sample of 5,954 patients 18 years or older who were admitted to East Surrey Hospital (ESH) ICU, which has an elective admissions rate of 3%, between 1 January 2005 and 31 October 2014. The total number of patients with sepsis was 941 compared with 5,013 without sepsis. We looked at mortality rates, APACHE II scores and length of stay on the unit.

## Results

From the beginning of 2005 to the end of October 2014, mortality rates in septic patients were 44.6% compared with 26.2% in nonseptic patients. Fisher's two-tailed test showed a significant difference (*P *< 0.0001) between the mortality in septic and nonseptic patients. There was a significant difference (Mann-Whitney) between APACHE II scores, with median scores of 18 and 13 in septic and nonseptic patients respectively. Septic patients had longer lengths of stay, with the mean and median 8.73 and 3.89 days respectively, compared with 4.90 and 2.5 in nonseptic patients. Septic patients made up 15.8% of all patients admitted to the ICU.

## Conclusion

Patients with sepsis admitted to ESH ICU made up a significant minority of patients admitted to the ICU. Septic patients had a 70% relative higher mortality rate compared with nonseptic patients. The mortality rate of 44.6% fits with previously quoted mortality rates in septic shock. Patients with sepsis had a significantly higher predicted mortality, recorded by their APACHE II score, which was statistically significant. This also meant they needed longer ICU care, with the average length of stay almost doubled.
